# Protein and Polysaccharide Fibers via Air Jet Spinning: Emerging Techniques for Biomedical and Sustainable Applications

**DOI:** 10.3390/ijms252413282

**Published:** 2024-12-11

**Authors:** Varsha Prahaladan, Nagireddy Poluri, Makara Napoli, Connor Castro, Kerem Yildiz, Brea-Anna Berry-White, Ping Lu, David Salas-de la Cruz, Xiao Hu

**Affiliations:** 1 Department of Physics and Astronomy, Rowan University, Glassboro, NJ 08028, USA; prahal55@students.rowan.edu (V.P.); poluri34@students.rowan.edu (N.P.);; 2 Department of Biomedical Engineering, Rowan University, Glassboro, NJ 08028, USA; 3 Department of Chemistry and Biochemistry, Rowan University, Glassboro, NJ 08028, USA; lup@rowan.edu; 4 Department of Biological and Biomedical Sciences, Rowan University, Glassboro, NJ 08028, USA; 5 Department of Chemistry, Rutgers University, Camden, NJ 08102, USA; ds1191@camden.rutgers.edu

**Keywords:** biopolymer, fiber, solution blow spinning, tissue engineering, drug delivery

## Abstract

Polymers play a critical role in the biomedical and sustainable materials fields, serving as key resources for both research and product development. While synthetic and natural polymers are both widely used, synthetic polymers have traditionally dominated due to their ability to meet the specific material requirements of most fiber fabrication methods. However, synthetic polymers are derived from non-renewable resources, and their production raises environmental and health concerns. Natural polymers, on the other hand, are derived from renewable biological sources and include a subset known as biopolymers, such as proteins and polysaccharides, which are produced by living organisms. These biopolymers are naturally abundant and offer benefits such as biodegradability and non-toxicity, making them especially suitable for biomedical and green applications. Recently, air jet spinning has emerged as a promising method for fabricating biopolymer fibers, valued for its simplicity, cost-effectiveness, and safety—advantages that stand out compared to the more conventional electrospinning process. This review examines the methods and mechanisms of air jet spinning, drawing on empirical studies and practical insights to highlight its advantages over traditional fiber production techniques. By assembling natural biopolymers into micro- and nanofibers, this novel fabrication method demonstrates strong potential for targeted applications, including tissue engineering, drug delivery, air filtration, food packaging, and biosensing, utilizing various protein and polysaccharide sources.

## 1. Introduction

Polymers have long served as a major component for biomedical uses, falling broadly into two categories: synthetic types and natural biopolymers [1,2,3]. Traditionally, synthetic polymers have been more commonly used as biomaterials due to their expansive and versatile nature [4]. However, these materials are manufactured using non-renewable resources, and many of them are not biodegradable [5]. Recently, there has been a growing interest in replacing synthetic polymers with natural and synthetic biopolymers as primary materials for biomedical and green applications [6,7,8]. Natural polymers refer broadly to materials derived from biological origins, including polysaccharides, proteins, and lignin, whereas natural biopolymers specifically denote macromolecules like proteins and polysaccharides formed by living organisms. These biopolymers offer unique advantages, such as intrinsic biodegradability, making them highly favorable for sustainable innovation [9,10]. The advantage of using biopolymers over synthetic polymers is that they are naturally derived or synthetically produced from renewable natural resources and are biocompatible and environmentally safe [2,11,12,13,14]. Moreover, biopolymers exhibit physiochemical properties comparable to their synthetic counterparts while also offering benefits such as biodegradability, natural abundance, non-toxicity, and combustibility [15,16,17]. The combustibility of biopolymers, while less significant in biomedical applications, is advantageous in other sectors such as packaging, energy generation, and environmental remediation, where controlled thermal degradation or energy recovery is required [18]. These qualities of biopolymers have led to increased applications in various fields, particularly in fiber production [19,20,21,22]. Fibers fabricated from biopolymers have unique structures with advantageous mechanical properties such as structural integrity, flexibility, and toughness [23,24,25,26]. Depending on the extraction method, biopolymers can display vastly varying properties, making them an extremely useful material class capable of serving a wide range of biomedical applications [27].

In recent years, researchers have increasingly turned to nanotechnology to enhance the versatility and functionality of biopolymers for biomedical use. Nanotechnology has gained popularity due to the unique and tunable characteristics of nanomaterials. Among these materials are biopolymer fibers, which can be structured in organized, porous fiber architectures similar to biological tissues [28,29]. Compared to bulk materials, these fibers provide greater surface area and higher surface energy, facilitating improved adhesion to cells, proteins, and drugs [30]. Advances in nanotechnology-based fiber fabrication methods now enable the production of fibers at both nanoscale and microscale levels, allowing for fine-tuning of mechanical properties such as fiber size, orientation, and porosity [31]. This degree of control has important applications, including the ability to influence cell behavior, making these fibers ideal for biomedical scaffolding [31,32,33].

A variety of methods can be employed to produce these fibers, including wet spinning, centrifugal jet spinning, self-assembly, melt blowing, plasma-induced synthesis, template synthesis, and electrospinning [28,34]. Among these, electrospinning is widely recognized for its ability to produce fibers with extremely small diameters, reaching down to the nanoscale [35,36]. Despite its simplicity, electrospinning has limitations, including low production yields due to slow solution ejection, operational and safety concerns associated with high voltages, and conductivity requirements that restrict the range of usable biopolymer solutions [37].

Air jet spinning (AJS), also known as solution blow spinning (SBS), is an alternative fiber fabrication method capable of producing fibers in the nano- to microscale range, similar to those generated by electrospinning. Air jet spinning (AJS) offers advantages such as higher production rates, enhanced safety by eliminating the need for high voltages, and compatibility with green solvents, making it more environmentally sustainable. For example, AJS has been successfully used to produce polycaprolactone (PCL) nanofibers for tissue engineering [38] and polyvinyl alcohol (PVA) nanofibers for filtration [39], achieving comparable quality with greater scalability. In their comparative study, Wojasinski et al. [32] evaluated the production of poly(L-lactic acid) (PLLA) nanofibers using both electrospinning and air-jet spinning (AJS). The study showed that AJS offers higher production rates and greater flexibility in processing parameters than electrospinning. This study also found that polymer solution concentration was the key parameter influencing the morphology and dimensions of the nanofibrous mats produced by both techniques. Specifically, an increase in polymer concentration led to an increase in average fiber diameter for both electrospinning and AJS.

Air jet spinning has also been shown to produce nanofibers that can be even smaller than those made by electrospinning [40]. This technique addresses many of the drawbacks associated with electrospinning, offering advantages in terms of polymer accessibility and cost-effectiveness (Table 1) [37]. Air jet spinning not only overcomes safety and equipment challenges but also improves production yield and allows for a broader selection of biopolymer raw materials and spinning solutions [41,42]. The fibers produced by air jet spinning (AJS) demonstrate structural uniformity, mechanical properties, and functional versatility comparable to those created via electrospinning but with significantly higher production rates, enhanced scalability, and cost-effectiveness due to the absence of high voltages [37,43,44].

**Figure 1:**
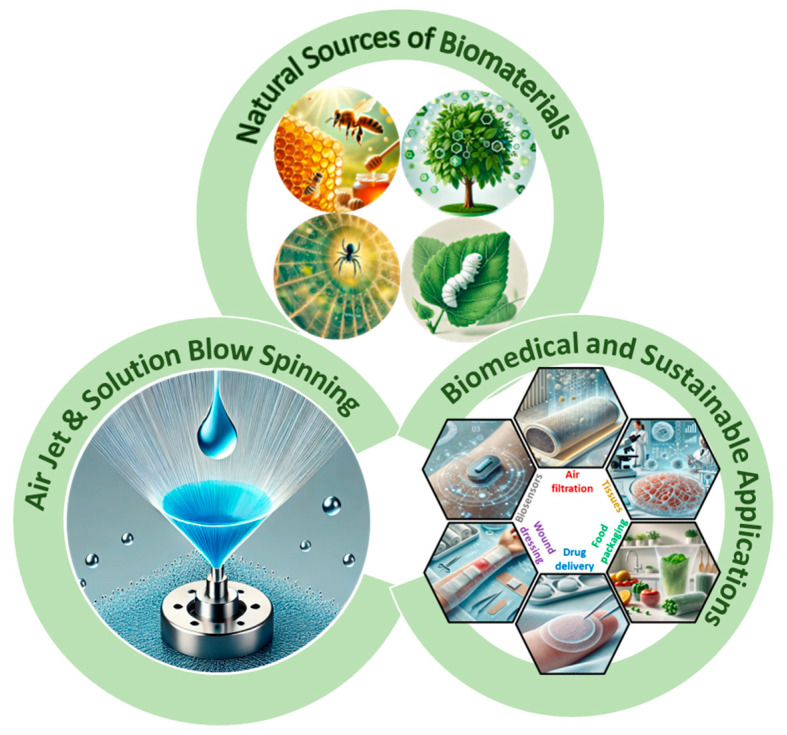
The schematic diagram illustrates the relationship between natural biomaterial sources, the air-jet/solution-blow spinning process, and their various biomedical and sustainable applications. (Cartoon pictures are generated by ChatGPT under a CC BY 4.0 license).

Figure 1 illustrates the key elements of this method, including its reliance on natural sources of biomaterials, the spinning process itself, and its various biomedical and sustainable applications, emphasizing its significance as a scalable and eco-friendly fiber production technique. Specifically, air jet spinning (AJS) utilizes pressurized gas to produce fibers from a biopolymer solution. A typical AJS setup includes a compressed gas source, pressure regulator, syringe, syringe pump, spraying apparatus with a concentric nozzle, and a collector [40,45]. This conventional configuration can be modified based on the specific characteristics of the polymer used and the requirements of the target material [42]. Such adaptability enables the fabrication of a wide range of materials, including fibers from synthetic polymers, composites, and bio-based polymers [46,47]. Studies have also highlighted the use of green solvent systems in AJS, reducing the reliance on toxic solvents and making the method suitable for biological applications [48]. The properties of AJS-produced fibers can be finely tuned to meet specific application needs. For example, adjusting the nozzle size allows for control over fiber diameter and orientation, creating fibers with varied structural and mechanical characteristics suited for different applications [49,50,51]. The versatility of materials and the adjustable system parameters make AJS a powerful tool for generating fibers with a broad spectrum of properties, enabling a diverse range of uses. The advantages of air-jet spinning as a fabrication method for biopolymers include cost-effectiveness, accessibility to raw materials, and the ability to precisely control product parameters, maximizing the utility of the resulting fibers across various biomedical and sustainable material fields.

**Table 1:** Comparison of key characteristics between electrospinning and air-jet spinning techniques.

Aspect	Electrospinning	Air Jet Spinning
Fiber formation	High voltage stretches the fiber [42,43,52,53]	Pressurized gas stream creates the fibers [42,43]
Solution conductivity	Plays major in elongation and thinner fibers [53]	Minor effect on elongation and diameter of fiber [53]
Fiber diameter (size)	Finer fibers [42]	Comparable to electrospun fiber diameter [42]
Production rate	Lower throughput [37]	Higher throughput [37]
Fiber uniformity	High fiber uniformity and control [42,54]	Moderate control over fiber uniformity [42,54]
Cost of setup	Expensive setup [54]	Relatively lower cost [42,55]
Mechanical strength	High toughness and flexibility [42]	High toughness and flexibility [42]
Application fields	Biomedical, filtration, composites, electronics [56]	Textiles [57], filtration [58], biomedical applications [59]

## 2. Potential Natural Materials

A wide range of protein and polysaccharide fibers can be fabricated through air jet spinning, supporting diverse applications across mechanical, biological, and chemical fields. Proteins, composed of long chains of amino acids and polysaccharides, made up of long-chain sugar molecules, each offer unique structural and functional benefits. This section examines various proteins—such as fish sarcoplasmic protein, collagen, gelatin, silk, zein, and soy protein—as well as polysaccharides, including starch, chitosan, and cellulose. Each biopolymer is introduced with background information, followed by a detailed overview of its inherent properties, highlighting the distinctive qualities that make these materials valuable for specific applications.

### 2.1. Proteins

Fish sarcoplasmic proteins (FSPs) are a type of muscle protein that is soluble in water [60]. These proteins are widely available from multiple sources, including Atlantic Cod (*Gadus morhua*) and fish industry-derived wastewater [61]. Fish muscle proteins fall into three main classes based on their solubility in water: connective tissue (stroma) proteins (3%), myofibrillar proteins (MP) (70–80%), and sarcoplasmic proteins (SP) (25–30%). While the MP and stroma proteins are water-insoluble, the SPs are readily soluble. Among the sarcoplasmic proteins in fish (FSP), several functional proteins can be found, including heme proteins like hemoglobin and myoglobin, as well as a variety of enzymes. These enzymes include aldolase, creatine kinase, phosphorylase, glyceraldehyde-3-phosphate dehydrogenase (GAPDH), proteases A and C, protease inhibitors, peroxidase, transglutaminase (TGase) and phospholipase. Additional proteins found in the FSP class are glycogen phosphorylase, fructose-bisphosphate aldolase A, triosephosphate isomerase B, beta-enolase, phosphoglycerate kinase, phosphoglucomutase, calmodulins and parvalbumins [62]. FSPs include enzymes such as hemoglobin and creatine kinase, which equip them with the ideal characteristics of a biomaterial for medical applications. Hemoglobin supplies oxygen and is known to promote wound healing. Creatine kinase aids in the production of ATP, supplying energy to muscle cells. Due to their enzyme-heavy makeup, varying the pH, temperature, and pressure of FSPs can change their mechanical properties [63]. Therefore, FSPs represent a low-cost and viable biomaterial for biomedical applications, particularly in tissue engineering [63].

Collagen, a fibrous protein, is a main structural component of the body’s extracellular matrix. Its abundance is present in all species of multicellular animals, making it an easily sourced material. Although 29 different types of collagens have been identified and studied, only types I, II, III, V, and XI are capable of forming fibers. These collagen fibers are composed of three α chains, with glycine regularly occurring at every third position in the amino acid sequence, while other amino acids, such as proline and 4-hydroxyproline, typically fill the remaining positions. Each of the 29 collagen types arises from a unique combination of three α chains selected from the 25 known distinct α chain conformations [64]. In the extracellular matrix, collagens provide tensile strength, promote cell adhesion and migration, and aid in tissue development [65]. It has excellent biocompatibility and biodegradability capabilities, making it an ideal material for tissue regeneration, most notably as a base for artificial tissue scaffolds [64]. Collagen’s structure is highly organized and three-dimensional, providing a network to house other components such as cells, proteins, or drugs. Additionally, the wide variety of collagen types, each with unique tissue distribution and functions, opens up possibilities for its integration with AJS and its applications in regenerative medicine [66].

Gelatin is a physiological protein sourced from denatured collagen, widely used in tissue engineering applications [67,68]. It is a cost-efficient biomaterial due to its natural abundance, minimal processing requirements, and ease of modification [68,69]. Gelatin is a biopolymer highly regarded as a base material in regenerative medicine due to its similarities to the extracellular matrix (ECM) and inherent biocompatibility. While gelatin shares similarities with ECM through its collagen origin, it lacks the fiber-collagen network that provides ECM with structural integrity. As a result, gelatin has limited structural strength and mechanical stability, making it unsuitable for standalone use in applications requiring durability [67]. Cross-linking is available to modify gelatin’s mechanical properties. Cross-linking can reinforce a gelatin base both physically and chemically, depending on the link used, and can, therefore, yield a huge variety of viable gelatin-based biomaterials to serve a specific function.

Silk fibroin is a fibrous protein-based biomaterial produced in nature primarily by spiders and silkworms [70,71]. Its structure is modular, featuring hydrophobic regions interspersed with hydrophilic groups. SF consists mainly of two chains: a hydrophobic heavy (H-) chain and a hydrophilic light (L-) chain, connected by disulfide bonds to form H-L complexes. This complex is stabilized by the glycoprotein P25, which helps maintain structural integrity. The H-chain is rich in glycine and includes repeating Gly-X dipeptide sequences, with hexapeptides such as Gly-Ala-Gly-Ala-Gly-Ser and Gly-Ala-Gly-Ala-Gly-Tyr making up about 70% of these sequences. The H-chain, L-chain, and P25 exist in a 6:6:1 molar ratio. The hierarchical structure of SF, especially its crystalline forms, silk I and silk II, significantly contributes to its unique biomaterial properties [72]. The benefits of using silk as a biomaterial include controlled biodegradability, good biocompatibility, flexibility, high strength, and non-toxicity [71,73]. A widely studied type of silk is derived from the *Bombyx mori* silkworms and consists of a fibroin core peptide surrounded by sericin [73]. Natural silks have a crystalline structure comprised of anti-parallel beta sheets, equipping them with favorable mechanical properties such as tensile strength and toughness [71]. It is known that silk’s structure and subsequent function are dependent on its amino acid composition. This knowledge enables processing techniques that optimize the mechanical and biological properties of silk while preserving the sequences that impart its beneficial qualities. The unique physical and chemical structure of silk makes it a valuable biopolymer, suitable for spinning into fibers using AJS for applications in tissue engineering and drug delivery.

Zein is the principal storage protein of corn. It is highly hydrophobic but is alcohol-soluble due to its lack of lysine and tryptophan [74,75]. Since it is derived from corn, it is widely available in nature at a relatively low cost. Material properties of corn zein protein include resistance to heat, water, abrasion and humidity [75]. Nanoparticles derived from corn zein protein have been shown to have optimal drug-loading capacities and drug-release profiles. The U.S. Food and Drug Administration (FDA) has classified corn zein as Generally Recognized as Safe (GRAS) for use in food applications, including as a film coating for pharmaceuticals [75]. Zein-based films have demonstrated biocompatibility, supporting both cell attachment and adhesion when cultured with various cell types [76]. Thus, corn zein protein is a popular choice for drug delivery applications, and with the capabilities of AJS, it holds promising potential for future applications in tissue engineering [76].

Soy protein isolate (SPI), a globular protein derived from readily available soybeans, this protein displays excellent characteristics relevant to biomedical applications, such as strong cell adhesion, support for cell metabolism, antibacterial properties, non-antigenicity, and ease of absorption. Soy protein is a promising candidate for promoting wound healing and tissue regeneration [77]. Compared to other natural proteins used in biomedical applications, soy protein offers notable advantages, including low cost, a plant-based origin, long shelf life, and stability. Additionally, its unique combination of properties, similarity to tissue components, and resistance to thermal degradation make it an appealing plant-derived macromolecule for use in the biomedical field. Fibers produced from soy protein, alone or combined with other proteins (e.g., silk) or green synthetic polymers like polyethylene oxide (PEO), hold significant potential for a variety of biomedical applications.

### 2.2. Polysaccharides

Starch, a plant-based polymer, is the second most abundant compound found in plants [78]. It is a polysaccharide that can be derived from almost all green plants, as well as from foods such as corn, various types of potatoes, rice, and wheat. Its ample sources in nature make it an extremely cost-efficient biopolymer. Other advantages of starch as a biopolymer include non-toxicity, renewability, biodegradability, and compatibility with other materials [79]. It is composed of two homopolymers known as amylose and amylopectin [80]. Both polysaccharides are composed of chains of α-(1,4)-linked D-glucose units, with branches formed through α-(1,6)-glycosidic bonds. While amylose is generally considered a linear molecule, some molecules do have a few branches. Both the linear and branched forms of amylose consist of long chains containing several hundred to a thousand glucose units. In contrast, amylopectin is highly branched and consists of shorter glucose chains by comparison [81]. Grouped together, these molecules form partially crystalline structures, which contribute to the mechanical properties of starch. Therefore, the structure of starch granules depends on the ratio of these molecules [79,80]. This knowledge allows many applications of starch, with various solution-blown structures capable of different functions dependent on molecular makeup.

Chitosan is a polysaccharide derived from chitin, the second most abundant biopolymer [82]. It is composed of varying amounts of (β1→4)-linked residues of N-acetyl-2-amino-2-deoxy-D-glucose (glucosamine, GlcN) and 2-amino-2-deoxy-D-glucose (N-acetyl-glucosamine, GlcNAc) [83]. Chitosan is found in crustacean shells, insects, and fungi [84]. It has extensive biomaterial applications due to its biological properties, including biocompatibility, non-toxicity, non-allergenicity, and biodegradability. It is also active in antibacterial, anti-tumor, and anti-inflammatory reactions [84]. Chitosan has a positive charge in the presence of acidic conditions, so it is insoluble in neutral and basic environments [85]. This attribute makes it extremely valuable in producing fibers from AJS for drug delivery applications.

Cellulose is a primary structural polysaccharide in plant cell walls and is extremely abundant in nature. It is found in trees, plants, fruits, bark, and leaves [86]. Its organic compound is a polysaccharide composed of a linear chain of several hundred to thousands of β-(1→4)-linked D-glucose units [87]. Various derivations of cellulose differ in their crystalline structure, giving rise to differing mechanical and physical properties [88]. As such, cellulose biomaterials have tunable properties that make them suitable for many different applications. It is a biocompatible material with low toxicity and sufficient elasticity and flexibility, which provides high mechanical strength [89]. The diverse forms of cellulose in nature, along with its ability to be tailored for specific structural and functional properties, make it a popular choice for biomedical applications and a viable candidate for air-jet spinning.

Alginate is a naturally abundant polysaccharide extracted from brown seaweed or produced through microbial fermentation by bacteria like *Azotobacter vinelandii* [90] and *Pseudomonas aeruginosa* [91]. It is biocompatible, non-toxic, hygroscopic, and biodegradable, making it ideal for human use [92]. Alginate is typically extracted using alkaline solutions, resulting in alginic acid, which can form salts like sodium, calcium, and zinc alginate. Chemically, alginate consists of β-1,4-D-mannuronic acid (M) and α-1,4-L-guluronic acid (G) blocks, with the M/G ratio affecting its properties and functionality [93,94,95]. Higher G content leads to rigid structures, while M-enriched alginates are more flexible and biocompatible [96]. Recently, alginate fibers have gained attention due to their tunable porosity, high surface area, and potential in various applications such as biosensors, wound dressings and drug delivery [97,98,99,100]. These diverse applications can enable alginate to be spun into fibers using air jet spinning.

Pectin is a low-cost and abundant heteropolysaccharide commonly used as a gelling, thickening, and stabilizing agent in food products [47,101]. While pectin has been successfully spun into nanofibers using electrospinning, Martin et al. introduced the first reported method of producing pectin-based nanofibers via the AJS technique [47]. Further research on pectin-based air-jet spun nanofibers needs to be explored, as pectin offers significant potential for use in many biomedical applications, such as wound healing [102] and biosensors [103].

## 3. Fabrication Methods

Air jet spinning (AJS), also known as solution blow spinning, is a fiber fabrication method developed in 2009 that uses compressed air to extrude a polymer solution into micro- or nanoscale fibers [37]. It combines the best aspects of electrospinning—a widely used method for nanofiber production that relies on high voltage but is limited in scalability and safety [104]—and melts blowing, which uses high-velocity hot air to produce microfibers but struggles to achieve nanoscale fibers [105], enabling controlled production of nano- and microfibers at ten times the typical production rate [106,107]. This technique is versatile, capable of producing fibers for various applications using a wide range of materials, including polystyrene (PS) [37], Polymethylmethacrylate (PMMA) [37], polylactic acid (PLA) [37], PLA blends, proteins [108], polyesters [108], co-spun cellulose acetate with poly(ethylene oxide) [109], and polyethylene-co-vinyl acetate) [110].

**Figure 2:**
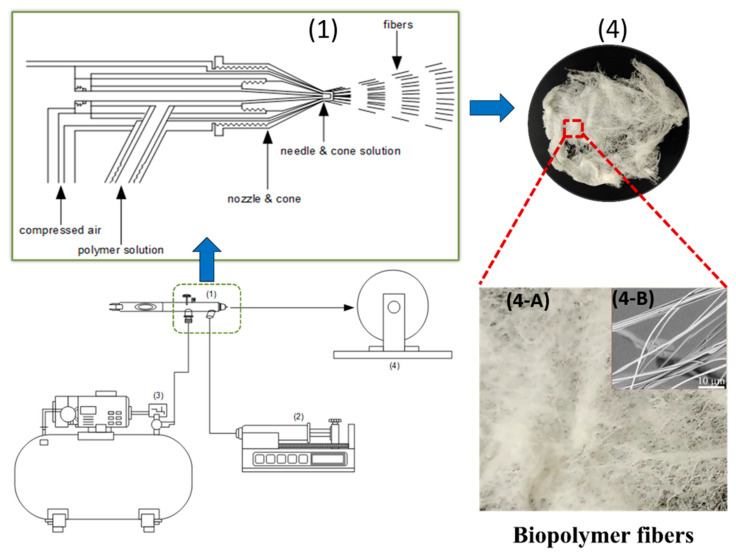
Diagram of a nozzle design utilized in air jet spinning. The left side illustrates a schematic diagram of an air-spinning setup, which includes (1) the nozzle and cone, (2) an injection pump with a syringe and blowing medium, (3) a compressor and pressure gauge, and (4) a rotating drum collector. The right side presents optical microscopy (4-A) and SEM (4-B) images of air-jet spun nanofibers, demonstrating the interconnected network structure and detailed fiber dimensions (scale bar: 10 μm). These images showcase the morphology of the corn zein fibers as an example [111]. (Reproduced with permission from Elsevier, © 2022).

**Figure 3:**
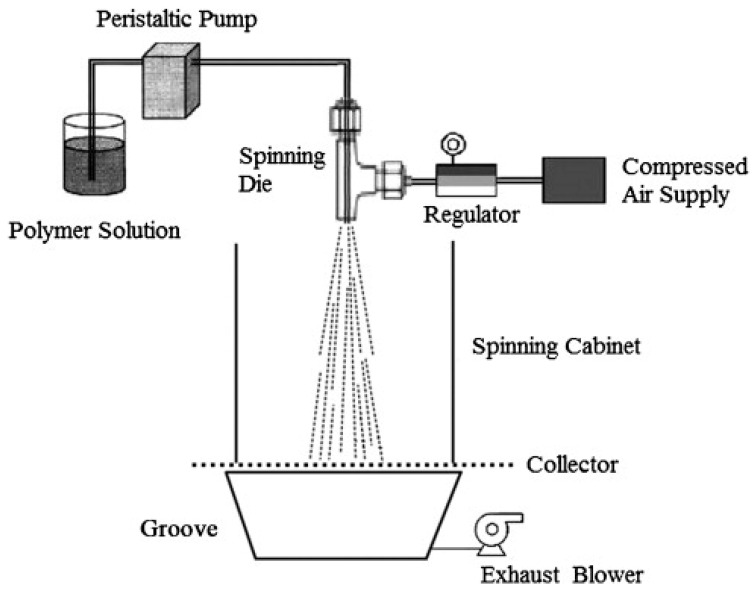
Schematic representation of a solution-blow spinning setup for the production of nanofibers from polymer solutions [112]. (Reproduced with permission from Elsevier, © 2012).

AJS uses two parallel concentric fluid streams of pressurized gas and polymer volatile solvents to create fibers. The generation of the fibers is dependent on concentration, molecular weight, gas pressure, viscosity of polymer solution, and solution flow rate [45]. The top left diagram in Figure 2 displays a nozzle design inside the air jet spinning. The air nozzle contains a needle which ranges from 0.2–0.7 mm in diameter. Polymeric gel solutions are drawn through needles equipped with heatable nozzles to produce micro- or nanofibers. The nozzle configuration pumps the polymer solution through high-pressure air. The nozzle’s geometry creates a low-pressure region that helps draw the solution into a cone shape. The polymeric gel solution is housed in the inner chamber, while the pressurized gas occupies the outer chamber. Gas velocity depends on the acceleration of air decompression, based on Bernoulli’s principle. The high-speed pressurized gas reduces pressure at the gas/solution interface, which stretches the polymer solution toward the collector. When air pressure is exceeded, a solvent droplet forms within the inner nozzle [37,111]. The SEM images in Figure 2(4-B) highlight the interconnected fiber network and smooth morphology of air-jet spun corn zein nanofibers, which are critical for biomedical applications like wound healing and drug delivery, as well as filtration due to their porosity and mechanical stability. The solution-blowing apparatus, as shown in Figure 3, features an annular spinning nozzle surrounded by a gas cavity, where a peristaltic pump regulates the polymer solution supply, and compressed air is controlled by a pressure regulator. Once the polymer solution exits the nozzle, it is transformed into fibers by gas streams and collected on a mesh-like collector, while a heating unit within the spinning cabinet aids solvent evaporation, which is then removed by an exhaust blower through a groove beneath the collector [112].

An airbrushing system is commonly used to create nanofibers during the AJS process. Airbrushing is 100 times less expensive and 10 times faster than electrospinning techniques. This method is also simpler and quicker to set up, as it involves fewer parameters that could impact reproducibility [65]. Additional advantages include improved safety, a faster deposition rate, greater versatility in shape and mobility, and the potential for directly spraying on wound dressings. The airbrushing procedure supports cell proliferation, differentiation, and adhesion comparable to electrospinning while achieving higher porosity [45]. The resulting fibers could be one to two orders of magnitude smaller than those produced by electrospinning [42].

Jet spinning is another technique used in AJS and is known for its high efficiency and flexibility [113]. Jet spinning is known to have a high production speed of up to 500 m/min, with speed control allowing for precise yarn evenness. Fibers produced by this method demonstrate low tendencies to pill or snarl, good abrasion resistance, and consistent evenness. The primary component of this system is the set of cylindrical channels through which fibers pass. Positioned along the channel axis are four nozzles that inject compressed air, creating a vortex airflow throughout the channel [113].

Various methods are used to prepare biopolymer solutions for air jet spinning. One approach involves downsizing silk fibers by applying high-intensity ultrasound to the silk solution before air jet spinning [77]. However, chemical treatments are more commonly used. These treatments selectively break hydrogen bonds without disrupting the nanofibril structures in natural biopolymers. Biopolymers can also be dissolved in different solvents, such as ionic liquids or acid-based solutions, to customize the final properties of fibers after AJS. For example, gelatin, prepared in powder form and dissolved in water, is set and denatured through gel electrophoresis, often using a lithium dodecyl sulfate buffer, before being fed through the air nozzle [114].

In conclusion, the use of high-turbulence gas improves the evaporation of the solvent during the process of air jet spinning. As a result of its nature, AJS deposits fibers directly onto the target surface, whether that be a collection plate or directly onto tissue and skin. The mechanisms for air jet spinning are similar to that of conventional airbrushing techniques. An outer nozzle is filled with a stream of compressed gas, whereas the inner nozzle is fed the polymer solution. The stream of gas creates a difference in pressure in the chamber, causing the polymer solution to elongate into a narrow stream in the direction of the gas, wherein the gas flow also causes the solvent of the stream to evaporate midair as it travels toward the collection target. This is similar to the method of using electricity to overcome surface tension and stretch the solution in electrospinning. The simplicity of air jet spinning means nozzles of various sizes can be used and interchanged rather easily, including standard airbrush painting nozzles. Air jet spinning can also be carried out using hand-held tools, as the airbrush itself is not inherently dangerous and is portable, especially when compared to the large and expensive apparatuses used for electrospinning. Blending the polymer solution with other types of materials can result in nanofibers with different properties and increased surface area [37]. Another benefit of air-jet spinning is the avoidance of using toxic solvents for the polymer solution. Due to the usage of an electric field instead of pressure differences, solvents of high dielectric constants are typical in electrospinning, and fiber deposition is a slow process. Thus, electrospinning is typically associated with greater safety hazards, toxicity, and environmental pollution due to the use of toxic solvents and high voltages [115,116,117,118].

A notable challenge in air jet spinning (AJS) is its higher energy consumption due to the use of compressed air, which can increase operational costs; however, these costs remain considerably lower than those of electrospinning, particularly in bench-scale settings, due to the simpler equipment setup and absence of high-voltage power supplies [119]. Future advancements in AJS are focusing on optimizing nozzle designs to enhance fiber uniformity and scalability while further reducing production costs. For instance, a study on Murata air-jet spinning demonstrated that specific nozzle configurations significantly influence yarn formation and quality, suggesting that tailored nozzle designs can improve fiber consistency and production efficiency [120]. In addition, integrating AJS with automated systems has shown great promise in improving reproducibility and expanding its applicability in fields such as tissue engineering and drug delivery. An automated solution blow spinning system, a variant of AJS, has successfully produced fibrous polymer scaffolds suitable for tissue engineering, underscoring the potential of automation to enhance process control and scalability [121].

## 4. Spinning Theory

There were a lot of parameters that needed to be considered for the air jet spinning, such as nozzle size, air pressure, and flow rate of solution to spin fibers. Pressure tests were conducted to evaluate the effect of suction pressure generated within the inner nozzle as a result of pressurized air exiting the outer nozzle. The manometric pressure (Δ*P*) is described by the following Equation (1):

Δ*P* = *ρgh* (1)

where *ρ* represents the density (kg/m^3^) at the measurement temperature, *g* is the gravitational constant (about 9.81 m/s^2^), and *h* is the column height (m).

The air velocity of the solution and the average stretching ratio between the air and the polymer solution $\left({\bar{V}}_{air}/{\bar{V}}_{ps}\right)$ can be calculated using the following Equation (2):

(2) $$\frac{{\bar{V}}_{air}}{{\bar{V}}_{ps}}=\frac{Q_{air}}{Q_{ps}}\cdot \frac{A_{in}}{A_{a}}=\frac{Q_{air}}{Q_{ps}}\left(\frac{{OD}^{2}}{{ID}^{2}-{OD}^{2}}\right)$$

where the average airflow velocity ${\bar{V}}_{air}\;$ at the nozzle exit, is given by the equation ${\bar{V}}_{air}\;$= *Q*_air_/*A*_a_. Here, *Q*_air_ is the airflow rate measured in liters per minute (L/min), and *A*_a_ is the annular area of the cross-section (in m^2^) between the inner and outer nozzles. The annular area is determined by the inside diameter (ID) of the outer nozzle and the outside diameter (OD) of the inner nozzle [116]. These equations were utilized by Ronelly et al. to calculate the air-jet spinning nozzle geometry and assess its effects on fiber morphology [116].

The dynamics of fiber formation in a biopolymer solution jet are determined by the balance between viscous forces, inertial-capillary forces, and the biopolymer’s relaxation time. This complex interaction is captured by two key dimensionless numbers: the Ohnesorge number (Oh) and the Deborah number (De) [117].

The Ohnesorge number (Oh) is defined as the ratio between the viscous time scale (*t_visc_*) (which is related to the fluid’s viscosity) and the inertial (or Rayleigh) time scale (*t_R_*) (which is associated with the fluid’s density and flow velocity). Oh is also associated with the capillary-thinning and break-up processes depending on both are determined by three-time scales: the viscous time scale (*t_visc_*), the polymeric time scale (*t_poly_~λ*), and the Rayleigh or an inertial time scale (*t_R_*) [117]. This can be expressed as Equation (3):

(3) $$Oh=t_{visc}/t_{R}=\frac{\eta_{0}l/\sigma }{\sqrt{\rho l^{3}/\sigma }}\sim \eta_{0}/\sqrt{\rho \sigma l}\;\;\sim \eta_{0}/\sqrt{\rho \sigma r_{0}\;\;\;}$$

In this context, *η*_0_ denotes the zero-shear viscosity, while *l* refers to the characteristic flow length scale, which, in this case, corresponds to the diameter of the nozzle in both the dimensionless numbers [117,122,123].

The previously defined Ohnesorge number gives an indication of the impact of viscous stresses, whereas the intrinsic Deborah number can be used to evaluate the significance of elastic stresses and the inertial time scale, which governs the inertio-capillary break-up of an inviscid jet. The Deborah Number can be represented as the ratio of the polymer relaxation time scale (*t_p_*) to the Rayleigh time scale (*t_R_*) [117]. To counteract the inertio-capillary forces, which are characterized by the Deborah number (De), the biopolymer’s relaxation time (*λ*) also plays a crucial role, as illustrated in the Equation (4):

(4) $$De=t_{p}/t_{R}=\lambda /\sqrt{\rho l^{3}/\sigma }\;\sim \;\lambda /\sqrt{\rho r_{0}^{3}/\sigma \;}$$

here, the Deborah number is influenced by solution density (*ρ*), surface tension (*σ*), and nozzle radius (*r*₀) which refers to the characteristic flow length scale (*l*), and appears in the denominator of both dimensionless numbers [117,122,123]. For Instance, In the study conducted by Srinivasan et al. [122], a relatively low Ohnesorge number was observed, suggesting that viscous stresses do not significantly influence fiber stabilization under the given conditions. Instead, the substantial increase in relaxation time and Deborah number points to the dominant role of viscoelastic effects. These effects slow down the jet breakup process, facilitating the formation of continuous fibers.

Fiber formation, morphology, and fiber diameter from a jet of biopolymer solution rely on the viscosity and entanglement of polymer chains [122]. Entanglement begins once the concentration reaches a critical level called the overlap concentration (c*). At this critical concentration, the polymer coils start to overlap, creating a semi-dilute solution. This overlap significantly increases the solution’s viscosity, a property essential for fiber formation because it helps to counteract surface tension. In this semi-dilute state, the interactions among these overlapping chains lead to an increase in viscosity. To determine c*, an Equation (5) was designed for biopolymers in a semi-dilute solution with a good solvent [45,118]:

c* = 6^3/2^*M*_w_/(8*N*_a_⟨*R*^2^⟩^3/2^) (5)

where *N*_a_ is Avogadro’s number, *M*_w_ is the average molecular mass of the biopolymer of interest, and the mean-square end-to-end distance of the polymer coils ⟨*R*^2^⟩ can be calculated by the following Equation (6) [118,124]:

⟨*R*^2^⟩ = *α*^2^*L*^2^*C*_∞_(2*M*_w_/*M*_0_) (6)

here, the Flory expansion factor (*α*), bond length (*L*), and characteristic ratio (*C_∞_*) are used to quantify the deviations from the ideal dimensions of polymer chains [124,125]. Srinivasan et al. [122] applied Equation (5) to estimate the overlap concentration of poly (methyl methacrylate) (PMMA) solution at various polymer molecular weights and to understand fiber formation, morphology, chain entanglement, and the viscoelasticity of polymer solution. Yu et al. [126] also explored the influence of fluid elasticity on fiber formation by using polymer solutions with varying elastic properties. A high degree of elasticity is shown to prevent droplet breakup, promoting uniform fibers over beads-on-string morphology [126]. Also, a study by Olivera et al. found that solutions with higher viscoelasticity created smooth, uniform fibers without bead defects. In contrast, solutions with lower elasticity resulted in fibers with varying diameters [127].

The AJS process is also influenced by the airflow rate, particularly at lower gas pressures [128], while extreme feed rates can lead to jet instability or nozzle clogging. Typical gas pressure ranges from 0.2 to 0.4 MPa, and the typical feed rates range from 20–1000 μL/min [128,129]. A smaller nozzle diameter generally results in finer fibers, and higher gas pressures contribute to narrow and consistent fiber distributions, which are ideal for precision-focused applications. However, excessive gas flow can cool the device due to expansion, impairing solvent evaporation and potentially causing fiber welding [128,130,131]. Achieving stable polymer jets requires an optimal balance of polymer flow rates and gas pressures. While the biopolymer stream is commonly maintained at room temperature, increasing its temperature can decrease viscosity or improve biopolymer solubility.

In the AJS process, the working distance and speed of the collecting drum also play a critical role in determining fiber morphology and quality. The working distance, defined as the gap between the nozzle and the collector drum, influences both the evaporation rate of the solvent and the behavior of the solution jet, impacting fiber formation. At shorter distances, fiber jets reach the collector quickly, retaining residual solvent and leading to film-like structures rather than discrete fibers. As the receiving distance increases, the jet experiences enhanced solvent volatilization and stretching, resulting in finer, well-defined fibers. However, excessive receiving distances can lead to jet dispersion, reducing fiber collection efficiency as fibers miss the collector. Thus, optimal conditions need to be identified for any polymer system to balance sufficient drying time and controlled jet deposition, finely tuning the receiving distance to optimize nanofiber quality and consistency in AJS applications [132,133]. The speed of the collecting drum also plays a significant role in determining fiber morphology. It is important to maintain a balanced rotational speed to ensure a consistent linear velocity, which helps optimize fiber dimensions [134]. In certain cases, depending on the type of polymer solution, a high collecting drum speed can also lead to the production of uniform fibers without beads [135]. All these parameters, such as air pressure, nozzle size, flow rate, viscosity of the solution, elasticity, working distance, and speed of the collecting drum, were equally important for any polymer system during the air jet spinning process to obtain fibers with the desired dimensions.

## 5. Biomedical and Sustainable Applications

Many biomaterials have garnered interest in air jet spinning, with sources including silk, soy, zein, gelatin, cellulose, chitosan, collagen, and starch. These organic biopolymers must be carefully processed to create materials suitable for a range of biomedical and sustainable applications, including tissue engineering, wound healing, drug delivery, air filtration and biosensing.

### 5.1. Tissue Engineering

Tissue engineering is a field within regenerative medicine focused on producing engineered tissues by combining cells, scaffolds, and biologics for implantation into the body to support tissue repair [136,137,138]. This process involves extracting cells from a tissue source, expanding them in culture, placing them onto a scaffold, and preparing them for implantation to create functional tissues or organs using materials derived from human, animal, or synthetic sources [139,140]. Scaffolds can be made from a wide range of materials that possess essential properties for successful regeneration, including biocompatibility, biodegradability, bioabsorbability, bioavailability, and non-cytotoxicity [141,142,143]. The shift from synthetic materials to the incorporation of biodegradable polymers has enabled the development of more biocompatible and biodegradable scaffolds in the field of tissue engineering [144].

Air jet spinning (AJS) has become a promising technique for creating nanofibrous scaffolds that effectively replicate the extracellular matrix (ECM), largely due to their large surface area relative to volume, which improves cell attachment and nutrient diffusion. Unlike other fabrication methods like electrospinning, AJS allows for greater scalability and enables the production of fibers with tailored characteristics. Recent studies have demonstrated the potential of AJS in tissue engineering. For instance, hybrid sol-gel inorganic/gelatin porous fibers fabricated via AJS exhibit enhanced bioactivity and mechanical properties, making them suitable for bone tissue engineering by supporting cell attachment and mineralization [145]. Similarly, AJS has been used to produce porous and aligned regenerated silk fibroin fibers that mimic the ECM, promoting cell adhesion, proliferation, and differentiation, which are critical for applications such as wound healing and vascular grafts [31]. A 2023 study by Zhang et al. introduced a double-stretching AJS technique to fabricate sub-100 nm nanofibers with improved scaffold performance for tissue regeneration, highlighting its potential for precise applications in regenerative medicine [146]. These AJS-generated nanofibrous scaffolds offer notable advantages by creating an optimal setting for tissue regeneration, making them a more adaptable and economical choice for use in tissue engineering.

Table 2 summarizes the biopolymer materials used for nanofiber production via air jet spinning, including details on pressure conditions, pretreatment methods, post-treatment methods, and biomedical applications. Gelatin and collagen, favored for biomedical uses, enhance cell adhesion and mechanical properties, especially when blended with pullulan or cross-linked with carbodiimide or glutaraldehyde. Chitosan and cellulose, ideal for air filtration and biosensing, benefit from green solvent dissolution or blending with biopolymers, with post-treatments improving antimicrobial and cell-proliferation properties. Silk, known for its strength and flexibility, is suitable for tissue engineering and drug delivery. Pretreatments like blending with anthocyanins boost wound healing, while ultrasound-assisted spinning enables structural modifications for targeted applications.

#### 5.1.1. Tissue Scaffolding

Tissue scaffolding has become a prominent area of research, particularly in the field of nanofiber technologies. Biopolymer-based nanofibers and their composites are attracting increasing attention due to their biocompatibility, sustainable production methods, and renewability. A notable study by Sett et al. introduced the first fish sarcoplasmic protein-based nanofiber created using the AJS method. Results from tensile tests, fiber diameter measurements, and porosity calculations demonstrated that this material could withstand mechanical forces and has good porosity. Coupled with its biocompatibility and natural bioactivity, this type of nanofiber shows significant potential as a tissue scaffold [61].

Expanding on composite scaffolding, Akentjew et al. developed a hybrid hydrogel/fiber tissue graft using a cell-laden methacryloyl gelatin-alginate hydrogel combined with PCL submicron fibers (a composite material combining a protein-based hydrogel a polysaccharide), designed to mimic and respond to the mechanical properties of human coronary arteries. The team successfully matched the mechanical characteristics of native coronary artery tissue, achieving the natural J-shape of the artery—a challenging feat for materials in this application. Additionally, the fibers can be easily customized to replicate other native blood vessels, showing promising results for the replacement of small-diameter blood vessels. The group also demonstrated the capability to encapsulate various cell types to enhance biological function [149].

**Figure 4:**
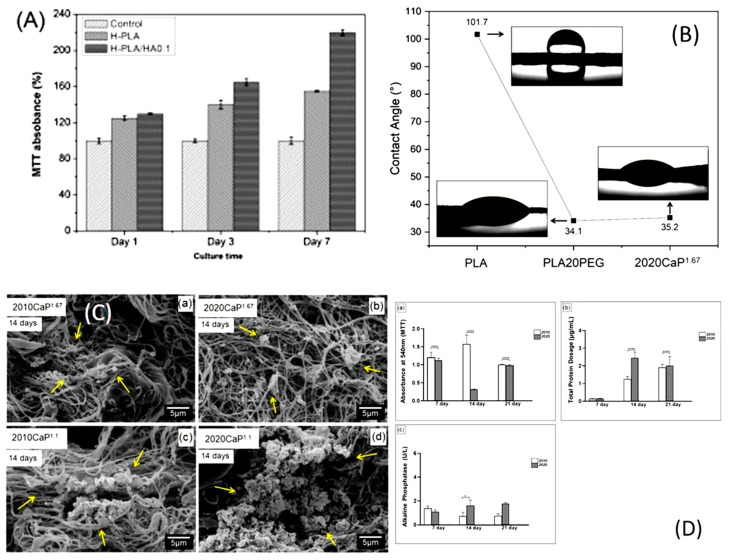
(**A**) MTT assay results showing the proliferation of MC3T3-E1 cells on PLA/HA nanofiber scaffolds over seven days, compared to a PLA control, indicating the scaffold’s support for cell growth [164]. (Reproduced with permission from Elsevier, © 2013) (**B**) Contact angle measurements for PLA, PLA20PEG, and 2020CaP^1.67^ scaffolds created via solution-blow spinning, demonstrating enhanced wettability with the incorporation of PEG and CaP. (**C**) SEM images of PLA/PEG/CaP scaffolds after 14 days in simulated body fluid, showing apatite deposition (marked with yellow arrows) in scaffolds with Ca/P ratios of 1.67 (**a**,**b**) and 1.1 (**c**,**d**). (**D**) Osteogenic indicators in MC3T3-E1 cells, including (**a**) MTT activity, (**b**) total protein content as a marker of cell proliferation, and (**c**) ALP activity, indicating osteogenic differentiation, were observed over 7, 14, and 21 days. Statistical significance is denoted by * (*p* < 0.05) and **** (*p* < 0.001) [165]. (Figures (**B**–**D**) are reproduced with permission from MDPI 2024, under the terms of the Creative Commons Attribution License).

Another application of air-jet spun biopolymer nanofibers was demonstrated in an in vitro study on cell proliferation and growth using a PLA/HA (hydroxyapatite) nanocomposite nanofiber (a bio-based polymer composite). The study showed that the PLA/HA scaffold promoted the growth and proliferation of MC3T3-E1 osteoblast-like cells for up to seven days, which is critical for early bone formation. Figure 4A displays the proliferation of MC3T3-E1 cells on a PLA/HA nanofiber scaffold over seven days, compared to a standard PLA control nanofiber. The scaffold also exhibited good mechanical properties, with tensile strength ranging from 23.8 to 5.5 MPa, depending on HA concentration. The percentage of elongation also varied with HA concentration, enabling possible modifications tailored to the specific requirements of the target bone structure [164].

In an effort to scale AJS technology, Tutak et al. adapted it into an airbrush-based method. They successfully sprayed several nanofibers, including PCL, demonstrating that this method could be applied to a wide range of polymers and potentially biopolymers. This approach holds significance for creating nanofiber molds of different organs and supporting stem cell differentiation, with promising applications in tissue engineering. Although the researchers primarily tested synthetic polymers and synthetic biopolymers, they compared the results to electrospun versions of these nanofibers and found that materials suitable for electrospinning could be effectively airbrushed with similar results [129].

Further research into multifunctional nanofibers was conducted by Bienek et al., which tested a chitosan/poly(lactic-co-glycolic acid)/polyethylene glycol (chitosan/PLGA/PEG) nanofiber (a polysaccharide polymer composite) to be used as a three-dimensional tissue scaffold. The study found that cell proliferation and antibacterial properties varied with the concentration of chitosan in the nanofiber. Higher chitosan concentrations improved antimicrobial properties but reduced cell proliferation. An optimal chitosan concentration of 1–2.5% was identified for balancing cytocompatibility and antimicrobial effectiveness. Also, the group was able to adhere and proliferate rat epithelial cells on the scaffold for up to 9 days [160].

Da Silva et al. investigated AJS scaffolds made from polylactic acid/polyethylene glycol/calcium phosphate (PLA/PEG/CaP) blends (a bio-based polymer composite) for potential use in bone tissue engineering [165], focusing on their wettability, mineralization, and interactions with cells. In Figure 4B, contact angle measurements reveal the fibers’ hydrophilic properties. Pure PLA scaffolds exhibited high hydrophobicity with a contact angle of 101.7° while incorporating 20% PEG (PLA20PEG) significantly lowered the angle to 34.1°, making the surface more hydrophilic. Adding 20% CaP further lowered the contact angle to 35.2°, indicating that CaP helps maintain increased wettability. Figure 4C shows the SEM images of the scaffolds after 14 days in simulated body fluid, showing apatite deposits (highlighted by yellow arrows). The SEM images also reveal a well-interconnected porous structure, which enhances nutrient and oxygen diffusion, creating a favorable environment for cell proliferation and tissue growth. The mineralization was more prominent in scaffolds with a Ca/P ratio of 1.1, which is largely due to the presence of β-TCP (β-tricalcium phosphate), which accelerates the mineralization process. Figure 4D shows the cellular response of 2020CaP^1.1^ (blend containing 20% *w*/*v* PLA/PEG and 20% *w*/*v* calcium phosphate with a Ca/P ratio of 1:1) and 2010CaP^1.1^ (blend containing 20% *w*/*v* PLA/PEG and 10% *w*/*v* calcium phosphate with a Ca/P ratio of 1:1), where the MTT assay results in panel (a) of Figure 4D indicates increased cell viability on the 2020CaP^1.1^ scaffold after 14 days. Total protein content also increased steadily, as shown in panel (b) of Figure 4D, reflecting cell proliferation and matrix production. Panel (c) of Figure 4D displays ALP activity, chiefly elevated in the 2020CaP^1.1^ scaffold, that again suggests an improved osteogenic differentiation. These results all together lay emphasis on the bioactive potential of PLA/PEG/CaP scaffolds for bone tissue regeneration [165].

#### 5.1.2. Cell Culturing

Biopolymer-based nanofibers offer unique advantages for cell growth compared to other methods. A notable example is the use of AJS biopolymer nanofibers in a cell culture study on a PLA/HA nanocomposite (a bio-composite) scaffold. This study demonstrated that the nanocomposite scaffold supported the growth and proliferation of MC3T3-E1 cells for up to seven days. Initially, cell growth on the first day was only slightly higher than in the control group. However, by the seventh day, cell proliferation had increased significantly, with cells evenly distributed across the scaffold. These findings highlight the potential of AJS biopolymer nanofibers for cell culture applications [164].

**Figure 5:**
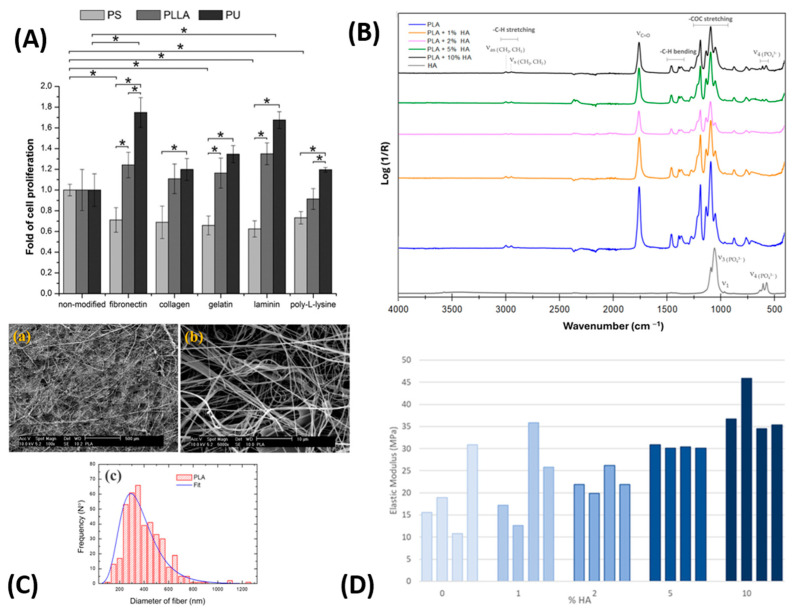
Various analyses of air-jet spun nanofibers for tissue engineering applications. (**A**) Impact of biopolymer coatings on the proliferation of human cardiomyocyte cells (H9C2) on PLA, PS, and PU nanofibers is shown, highlighting significant improvements after 7 days compared to uncoated fibers. Protein modifications such as fibronectin, collagen, gelatin, laminin, and poly-L-lysine enhanced cell proliferation, with PU nanofibers modified with poly-L-lysine demonstrating the highest fold change. Asterisks (*) indicate statistically significant differences (*p* < 0.05) [150]. (Reproduced with permission from Elsevier, © 2017). (**B**) ATR-FTIR spectra for PLA/HA samples, highlighting characteristic absorption bands at different HA concentrations. (**C**) SEM images of pure PLA nanofibers captured at (**a**) 100× and (**b**) 5000× magnifications, showing a fibrillar morphology with uniformly distributed fibers; (**c**) Histogram of fiber diameter distribution reveals a mean fiber diameter of 370 ± 140 nm, emphasizing consistent fiber formation. (**D**) Elastic modulus of PLA/HA composites as a function of HA content, showing increased stiffness with higher HA concentrations. PLA with 10% HA exhibited the highest modulus, demonstrating enhanced mechanical properties [166]. (Figures (**B**–**D**) are reproduced with permission from MDPI 2024, under the terms of the Creative Commons Attribution License).

Tomecka et al. created nanofiber mats made of PLLA and PU, which were further coated with collagen, gelatin, poly-L-lysine, fibronectin, and laminin (a bio-composite with protein coatings) to examine their effects on cell proliferation. The study showed that these mats successfully mimicked an in vivo environment for cardiac cell culture by enabling cell alignment similar to muscle tissue and muscle fibers. In addition, cell proliferation on the coated nanofiber varied based on the base material and the specific cell type being tested. For human cardiomyocyte cells, PLLA-based nanofibers performed best, with all coatings enhancing cell proliferation compared to uncoated PLLA nanofibers. Among the coatings, fibronectin on PLLA showed the highest average cell proliferation for human cardiomyocyte cells (Figure 5A) [150].

Magaz et al. evaluated AJS for regenerating silk fibroin nanofibers. Their findings demonstrated that AJS is a highly effective method for producing silk fibroin nanofibers (a protein polymer), resulting in aligned, nonporous fibers suitable for cell culturing applications. The primary aim of the study was to establish AJS as a viable technique for silk fiber manufacturing. By measuring fiber viscosity through rheology and assessing mechanical strength, the researchers confirmed that AJS-produced silk fibers show promise for biomedical applications [31].

Klaas et al. created a 3D nanofiber scaffold made of mammalian gelatin and glucose (a protein and polysaccharide blend) to mimic the natural extracellular matrix and main functional aspects of human liver tissue. To prove this, they showed they were able to culture human primary hepatocytes in the porous 3D scaffold. This method for mimicking the human liver offers improvements over traditional methods, such as animal models and 2D cell cultures, by providing a cheaper, faster, and more consistent approach. Its biomedical application includes the potential for long-term in vitro studies on liver-targeted pharmaceutical drugs using a more realistic model [68].

In another study, Gonzalez-Benito et al. [166] explored the development of polylactic acid/hydroxyapatite (PLA/HA) nanocomposite fibers (a bio-based composite) via AJS for tissue engineering applications. They investigated how varying amounts of HA affect the structural, thermal, and mechanical properties of these composites. As shown in Figure 5B, ATR-FTIR spectra reveal distinct absorption bands from both PLA and HA, indicating that HA integrates smoothly into the PLA matrix without forming new chemical bonds. Figure 5C displays SEM images of pure PLA fibers, showing a randomly distributed fibrillar structure with slight increases in fiber thickness corresponding to the rise in HA concentration. Analysis of fiber diameter distribution (Figure 5C) suggests that HA content subtly influences fiber size and formation. It also suggests that uniform and controlled fiber diameters provide an ideal scaffold for enhanced cell attachment and proliferation. Furthermore, mechanical tests in Figure 5D demonstrate that increasing HA content leads to a rise in the elastic modulus, indicating enhanced material stiffness while preserving flexibility, particularly in fibers with larger diameters. In summary, these findings highlight that by adjusting HA content, fiber diameter, and crystallinity, PLA/HA composites can be effectively tailored for biomedical applications.

A study by Victor et al. [161] explores the creation of air-jet spun polylactic acid/chitosan (PLA/CS) (a bio-based polymer and polysaccharide composite) and polyvinyl alcohol/chitosan (PVA/CS) (a synthetic polymer and polysaccharide composite) blends as 3D fibrous structures for tissue regeneration [161]. These polymer fibers are noted for their high porosity, biocompatibility, and tissue-like morphology. To address the challenges of stabilizing high-chitosan content fibers without cross-linking agents, the researchers utilized solution blow spinning with eco-friendly solvents in a heated air environment. Characterization of these fibers revealed that increasing chitosan content led to higher solution viscosity, which limited fiber formation but resulted in interconnected, highly porous fibers. Fiber diameter measurements ranged from 481 to 637 nm for PVA/CS and 1276 to 2050 nm for PLA/CS blends. The chitosan-rich fibers also demonstrated enhanced hydrophilicity, porosity, swelling, and in vitro biodegradability, underscoring their potential as promising scaffolds for tissue engineering.

#### 5.1.3. Wound Dressing

**Figure 6:**
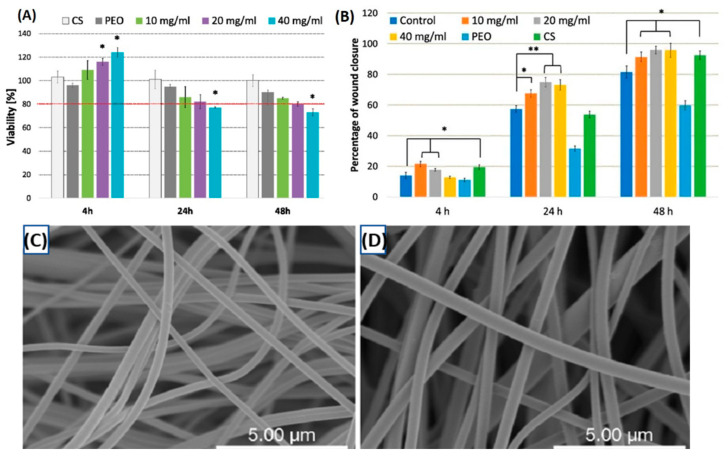
(**A**) Cell viability (relative to untreated control cells) of fibroblast cells exposed to various concentrations (10, 20, and 40 mg/mL) of 10% CS/PEO nanofibers alongside pure CS and pure PEO controls, over incubation periods of 4, 24, and 48 h. Significant differences in cell viability are marked by * for *p* ≤ 0.05. (**B**) Percentage of wound closure over a 48-h period from a scratch assay illustrating cellular migration and proliferation. The assay compares control cells with cells treated with CS/PEO nanofiber extracts (10% concentration) at 10, 20, and 40 mg/mL, with pure PEO and pure CS as additional controls. Significant differences in wound closure are denoted by * for *p* ≤ 0.05 and ** for *p* ≤ 0.01 when compared to untreated cells, demonstrating the effectiveness of the extracts in promoting cell migration and wound healing. (**C**,**D**) representative SEM images of CS/PEO nanofibers produced from 8% (**C**) and 10% (**D**) polymer blend solutions, showing the structural and morphological differences. Scale bar: 5 µm [167]. (Reproduced with permission from MDPI 2022, under the terms of the Creative Commons Attribution License).

Air jet spinning of biopolymer nanofibers shows promise for wound dressing applications due to their modifiable pore sizes and biocompatibility. Szymanska et al. conducted a study on air-jet spun chitosan/polyethylene oxide (CS/PEO) (a polysaccharide composite) nanofiber mats, highlighting their potential in wound healing and skin regeneration [167]. Scanning electron microscopy (Figure 6C,D) revealed a highly porous, three-dimensional structure with evenly distributed smooth fibers with average diameters of 200 nm and 260 nm for 8% and 10% CS/PEO formulations, respectively [167], which is critical for maintaining moisture at the wound site. The observed uniform morphology and absence of beads are critical for maintaining moisture at the wound site, and such properties align with the structural requirements for effective wound-healing materials. The researchers further evaluated the hydrophilicity, bio-adhesion, exudate absorption, and biocompatibility of the nanofibers, finding that the fibers retained moisture well, adhered strongly to skin tissue, and absorbed fluids at levels comparable to commercial dressings. In addition, the CS/PEO nanofibers were non-toxic to fibroblast cells, confirming their suitability as bioactive wound dressings for effective skin repair. The MTT assay (Figure 6A) shows that CS/PEO nanofibers exhibit admirable biocompatibility, with lower concentrations (10 and 20 mg/mL) supporting cell viability over the 48-h period. A slight decrease in viability was observed at the 40 mg/mL concentration, suggesting a dose-dependent effect on metabolic activity. This outcome is in line with the observed quickening of wound healing in the scratch assay, reinforcing the potential of CS/PEO nanofibers to promote skin repair while maintaining cytocompatibility. An in vitro scratch assay confirmed their wound healing capabilities over a 48-h period, as shown in Figure 6B. The CS-based extracts at 10, 20, and 40 mg/mL suggestively encouraged wound closure, with noteworthy effects in the early hours. The 10 mg/mL extract and pure CS-induced rapid cell migration, while pure PEO delayed closure. After 24 h, higher concentrations of CS/PEO extracts accomplished nearly 80% scratch closure, accentuating CS’s ability to accelerate early-stage wound healing. These results support the role of chitosan in reducing inflammation and enhancing fibroblast proliferation, which is essential for wound repair and regeneration.

Baek et al. created a PCL-based nanofiber combined with catechin and gelatin (a protein and synthetic polymer composite) for wound healing applications. Their results showed that this combination exhibited excellent biocompatibility, good healing properties for wounds, the ability to protect the wound from external factors, and a great antioxidant ability to better protect and heal the wound. The group tested cell proliferation on the nanofiber and found that with a higher concentration of (+)-catechin (10%), cell proliferation matched the control, indicating minimal cytotoxicity. They also measured NO₂ production at various levels of (+)-catechin, finding that the 10% concentration resulted in the lowest NO₂ production among the treatment groups, suggesting the best antimicrobial properties [151].

Tien et al. developed a deacetylated chitosan nanofiber (a polysaccharide composite) for wound healing applications. By deacetylating the chitosan and adding PEO to the solution before nanofiber production, they improved the fiber’s mechanical properties. The resulting nanofiber scaffold exhibited sufficient toughness and strength in both wet and dry states, making it suitable for potential wound dressing applications. Also, the scaffold supported cell proliferation and viability, successfully sustaining NHDF cells, further reinforcing its potential for wound healing applications [159].

Snari et al. generated a SP/PVA/ZnO nanocomposite (a protein composite) nanofiber for biomedical applications. The nanofiber has good antibacterial properties for both gram-negative and positive bacteria, making it a promising material for wound dressing. The nanofiber was tested using a saline solution, which demonstrated its capacity to absorb fluids, suggesting effectiveness in absorbing wound exudate. To assess its antibacterial capabilities, Snari et al. examined inhibition zones for two gram-positive bacteria (*B. pumilus* and *S. aureus*) and two gram-negative bacteria (*K. pneumoniae* and *E. coli*). The addition of ZnO in the PVA/SP nanofiber increased inhibition zones by 11–18% compared to nanofibers without ZnO [168].

Lorente et al. created a PCL/collagen (a protein composite) nanofiber for skin regeneration and wound dressing applications. The study measured cell viability and proliferation of HaCat epithelial cells. Results showed that after 7 days, cell viability was able to stay above 85%, and the cell proliferation was around 300–400%. Compared to a control group with only PCL (no collagen), all collagen-containing samples showed significantly higher cell viability and proliferation after 7 days [43].

### 5.2. Drug Delivery

**Figure 7:**
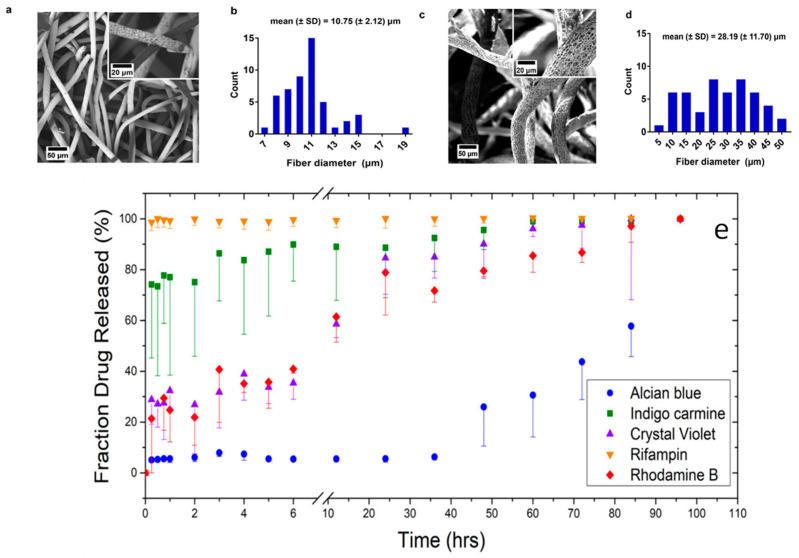
(**a**,**c**) SEM images of porous silk fibrous nanofibers spun at different concentrations (**a**) 27% *w*/*v*; (**b**) 30% *w*/*v*; (**b**,**d**) their corresponding fiber diameter measurements [28]. (Reproduced with permission, © 2018 American Chemical Society, licensed under CC BY.) (**e**) Drug release profiles of a silk nanofiber compared with different model drugs, illustrating how various release profiles can be achieved depending on the drug type carried by the nanofiber [125]. (Reproduced with permission from MDPI 2021, licensed under CC BY 4.0).

AJS biopolymers are promising for drug delivery applications due to their stability and adjustable pore sizes. DeFrates et al. utilized corn zein protein (a protein polymer) to form nanofibers as a potential method for drug delivery. Their results demonstrated that these nanofiber mats could store and sustainably release multiple model drugs, including rhodamine, alcian blue, rifampin, indigo carmine, and crystal violet. They also tested cell proliferation using human embryonic kidney cells. After 72 h, the zein fibers showed a significant increase in cell density compared to a blank surface [111]. Another study examined air jet-spun corn zein (a protein polymer) nanofibers incorporated with sodium citrate for potential use in the treatment of diabetic ulcers. The results indicated that the nanofibers effectively carried sodium citrate and released it at a controlled rate. To further demonstrate the advantages of using zein nanofibers, the study found that the drug did not degrade at high temperatures due to the fiber’s good thermal stability. In addition, human embryonic kidney cells were also successfully proliferated on the nanofibers [156]. Bonan et al. developed a PLA/PVP (a bio-based composite) nanofiber mat encapsulating copaiba oil for drug delivery. Their findings showed that the release rate could be adjusted based on the PLA/PVP blend used in AJS. They also demonstrated that air-jet spinning nanofibers could control the release of volatile agents, like copaiba oil. Additionally, they tested the antimicrobial activity of the nanofiber and observed that increasing the concentration of PVP enhanced the antimicrobial activity of fibers [33]. Magaz et al. produced regenerated silk fibroin (a protein polymer) nanofibers using the AJS method. Their study demonstrated that these nanofibers could be fabricated with 3D interconnected pores and might be suitable for controlled drug release applications. The pores were analyzed using SEM, ATR-FTIR, and WAXD tests, which provided insights into fiber diameters, pore quantity, secondary structure fractions, and fiber density (Figure 7a–d) [31]. The SEM images reveal a well-organized porous structure with varying fiber diameters that significantly influence drug release kinetics, making the fibers adaptable for diverse therapeutic applications. Gough et al. conducted a study on silk nanofibers for potential drug delivery applications. Using model drugs such as rhodamine B, rifampin, indigo carmine, crystal violet, and alcian blue, they examined drug release profiles based on various properties, including molecular weight, solubility, charge, and hydrophobicity. The data showed that the nanofibers could achieve controlled release profiles, successfully delivering the model drugs. The release rate could be adjusted by modifying the geometry of the material, such as using films versus nanofibers (Figure 7e) [156].

A study by Liu et al. explored the incorporation of cinnamaldehyde, a natural antimicrobial, into fish skin gelatin (FSG) nanofibers using solution blow spinning (SBS) [169]. Higher cinnamaldehyde ratios increased particle size, viscosity, and nanofiber diameter, but it was observed that cinnamaldehyde was partially lost during the solution blow spinning process and was predominantly distributed on the surface of the resulting nanofibers. The nanofibers exhibited antibacterial activity against *E. coli* O157:H7, salmonella typhimurium, and listeria monocytogenes, with stronger effects than cast films. Lower temperatures improved cinnamaldehyde retention, with 10% cinnamaldehyde nanofibers showing the best stability over eight weeks. The findings suggest that SBS-prepared FSG nanofibers can effectively deliver antimicrobials. Szymańska et al. created a vaginal delivery platform using a nanofibrous mat made of chitosan (CS) and poly(ethylene oxide) (PEO) via solution blow spinning (SBS), designed for the controlled release of tenofovir disoproxil fumarate (TDF) to prevent sexually transmitted infections [170]. The physicochemical analysis showed even drug distribution throughout the mat and indicated interactions between TDF and the polymers. Safety evaluations using human vaginal epithelial cells (VK2-E6/E7) confirmed the mat’s compatibility. Antiviral tests revealed that the mat effectively inhibited HSV-2, with SBS not affecting the drug’s activity. The mat’s mucoadhesive and swelling properties were influenced by vaginal pH, which impacted drug release. Faster drug permeation was observed at pH 5.0, suggesting improved TDF absorption at higher pH levels.

### 5.3. Other Green Applications

#### 5.3.1. Air Filtration

Porosity control in AJS makes it a promising method for creating biopolymer-based nanofibers for air filtration. Sett et al. developed nanofiber mats using fish sarcoplasmic protein (FSP) (a protein polymer) for air filtration applications due to its effective porosity control. To assess porosity, the team analyzed pore size distribution and frequency at varying concentrations of FSP and nylon. They found that the lowest fiber diameter, 213 nm, occurred at 0% FSP, with fiber diameter and porosity increasing as FSP concentration rose [61]. Dadol et al. developed polyacrylonitrile/cellulose acetate (a polysaccharide composite) nanofibers with strong filtering and adsorption properties that are suitable for air filtration applications. By incorporating polyacrylonitrile, they were able to increase the concentration of cellulose acetate in the nanofiber, facilitating the spinning process. The study demonstrated that by adjusting pressures during fiber formation with FBS, they could increase cellulose acetate concentration, thereby expanding the fiber diameter range and introducing more beads into the fiber matrix [162]. Eticha et al. investigated biodegradable gelatin (a protein polymer) nanofiber filters produced using electrically assisted solution blow spinning (ESBS) to explore sustainable alternatives to synthetic filters that contribute to environmental pollution. The study examined the effects of gelatin concentration, air pressure, and electric voltage on the average fiber diameter (AFD) and average droplet area (ADA). Gelatin concentration was found to have a substantial impact on both AFD (98.27%) and ADA (56.26%), with fiber diameters ranging from 93.86 nm to 250.13 nm. The optimized filter (G-12120) achieved a 90.5% filtration efficiency for 0.3 µm particles and a pressure drop of 225 Pa, qualifying it as an E10 filter according to EN 1822 standards [171].

#### 5.3.2. Food Packaging

**Figure 8:**
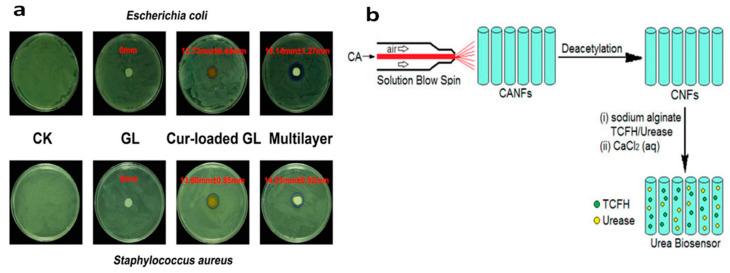
(**a**) Inhibition zone of *E. coli* on the multilayer nanofiber composed of polycaprolactone, curcumin-loaded gelatin, and polycaprolactone [113]. (Reproduced with permission from Elsevier, 2022, licensed under CC BY 4.0.) (**b**) Process of converting cellulose acetate into a urea biosensor [97]. (Reproduced with permission from MDPI 2021, licensed under CC BY 4.0).

In food packaging, the strength and antimicrobial activity of biopolymer-based nanofibers make them a promising alternative. Shen et al. developed gelatin and zein-based nanofibers (protein polymers) specifically for food packaging applications [153]. The biomedical significance of this study lies in demonstrating that these nanofibers can carry and release the antifungal agent natamycin, thereby imparting antimicrobial properties. The researchers examined various characteristics of the nanofibers to assess their potential effectiveness, including loading capacity and encapsulation efficiency of natamycin based on the drug mass incorporated into the fibers. The lowest amount of drug tested, 0.01 g, achieved the highest encapsulation efficiency at 94.78%, while the largest amount, 0.2 g, resulted in the highest loading capacity at 16.16%. For nanofiber’s antimicrobial activity, the study found that higher concentrations of natamycin in the nanofibers led to larger inhibitory diameters. For *Alternaria alternata*, inhibitory diameters increased from 22.0 mm at 0.01 g of natamycin to 69.98 mm at 0.2 g. Similarly, for *Botrytis cinerea*, inhibitory diameters rose from 28.97 mm to 63.46 mm as natamycin concentrations increased [153]. Cai et al. developed a multilayer nanofiber film composed of polycaprolactone and gelatin for the controlled release of curcumin. The curcumin release demonstrated antibacterial activity against *S. aureus* and *E. coli*, with inhibitory diameters of 12.73 mm and 13.60 mm, respectively. This antibacterial property, combined with the enhanced mechanical strength of the fibers, suggests promising potential for food packaging applications. When comparing the mechanical properties, the multilayer film exhibited 9 times the tensile strength of curcumin-loaded films alone, significantly improving its structural integrity (Figure 8a) [113].

Yang et al. developed Gelatin/PA66 composite nanofiber films for food packaging using solution blow spinning (SBS) [172]. Incorporating nylon 66 (PA66) enhanced the mechanical properties, water vapor resistance, and solvent resistance of the gelatin films. The composite films exhibited a significant increase in elongation and tensile strength while their water vapor permeability was reduced. The results demonstrate that SBS is an effective method for producing nanofibrous films, with PA66 enhancing the properties of gelatin for food packaging applications. Oliveira et al. investigated smart nanofiber nonwovens for detecting food spoilage by incorporating anthocyanin-rich extracts from purple cabbage and sweet potato into a zein/gelatin matrix using solution-blow-spinning [158]. The extracts reduced solution viscosity and nanofiber diameter, with the finest fibers formed at higher concentrations. The nonwovens exhibited improved mechanical, thermal, and water properties, along with antioxidant capabilities, and displayed visible color changes in response to pH and ammonia vapor. These materials were tested with milk and fish fillets, showing color changes that signal spoilage, highlighting their potential in sustainable food packaging.

#### 5.3.3. Biosensors

Chronic liver failure is a global health issue in which metabolic waste accumulates in the bloodstream, potentially leading to fatal outcomes. El-Newehy et al. developed a cellulose-based nanofiber (a polysaccharide polymer) composition (cellulose nanofiber combined with tricyanofuran hydrazone and urease) as a biosensor for urea detection aimed at assisting in the early detection of chronic kidney failure. The biosensor utilizes a tricyanofuran (TCF) acceptor paired with a hydrazone donor to form a TCFH probe, which is integrated into the nanofiber. This biosensor detects ammonia (NH₃) generated from the reaction between urea (CO(NH₂)₂) and urease. To evaluate the sensitivity of the biosensor, the study tested solutions with unknown urea concentrations. By referencing a calibration curve, they observed that the absorbance intensity of the nanofiber changed proportionally, yielding a linear response to urea concentrations ranging from 200 ppm to 1400 ppm (Figure 8b) [97]. Saores et al. developed biosensors using polylactic acid (PLA) fibers produced by solution blow spinning, designed for immobilizing an anti-p53 active layer to detect the p53 biomarker [173]. The fibers were deposited for 60 s, achieving a detection limit of 11 pg/mL using electrical impedance spectroscopy. The biosensors demonstrated high sensitivity, capable of detecting p53 in patient samples, with different p53 concentrations distinguishable using the IDMAP technique. The biosensors’ selectivity and sensitivity were attributed to the interaction between the active layer and p53, confirmed through infrared spectroscopy analysis. The successful immobilization of the active layer shows the potential of this fiber-based approach for other biosensor applications.

## 6. Transition from Laboratory Scale to Industrial Scale

Many biopolymers have been successfully spun at laboratory scales for various biomedical applications. However, transitioning from bench-scale to industrial-scale air jet spinning of biopolymers revealed several challenges, including dripping, capillary instability, and nozzle clogging. These issues, caused by low viscoelasticity at higher air supply temperatures, were observed by Kolbasav et al. [174] in their works on solution-blown soy/PEO blends. The resulting problems, such as droplet formation, fiber bundling, and bead formation, made it difficult to spin nanofiber mats. To address these challenges, the researchers optimized the experimental setup by employing 600 kDa PEO solutions, which enhanced viscoelasticity, stabilized the spinning process, and enabled the production of continuous fibers. Additionally, smaller spinnerets were used to reduce roping and fiber fusion, resulting in more uniform nanofiber mats. Successful mats were produced with PEO/soy protein blends, which mitigated dripping and fly formation. The resulting fibers exhibited consistent diameters (500~600 nm) with uniform distribution. This work demonstrated that proper solution composition and process optimization are crucial to overcoming the limitations of industrial-scale biopolymer spinning [174].

Similarly, Jin et al. developed a method for the large-scale, high-speed production of mullite fiber cotton at the industrial level through an air heating-solution blow spinning technique, achieving speeds ranging from 6 to 60 mL/h [175]. The researchers tested various aluminum sources to create spinnable Al-Si precursor solutions and optimized the solution blow spinning apparatus with a hot air injection system for efficient vaporization. In addition, key parameters such as solution viscosity, spinning rate, and spinning distance were optimized to control fiber diameter and morphology. After sintering at 1200 °C, the mullite fibers exhibited excellent thermal stability, insulation properties, and filtration efficiency. This approach promises cost-effective, high-quality mullite nanofibers for industrial applications.

Furthermore, Jia et al. highlighted several challenges in the solution blow spinning (SBS) process, including environmental concerns due to toxic solvent evaporation, nozzle blockages in multi-needle SBS equipment, and fiber bundling caused by the absence of electrostatic repulsion [50]. To address these issues, Jia et al. suggested developing eco-friendly spinning solutions with low-toxicity solvents, exploring needle-free SBS technology to prevent nozzle problems, and combining SBS with electrospinning to produce more uniform fibers. Jia et al. also recommended using natural materials to reduce environmental impact and urged improvements in SBS equipment, such as redesigning spinnerets, optimizing airflow channels to reduce energy consumption, and enhancing solvent recovery. Ensuring raw material consistency was identified as essential for maintaining fiber quality and advancing SBS technology in the fiber materials industry.

## 7. Conclusions

Nanofibers provide a unique and adaptable platform for developing advanced materials with applications spanning biotechnology, environmental sustainability, and healthcare. Although various materials can be used to create these fibers, biopolymer nanofibers have emerged as a sustainable and effective choice, positioning them as ideal candidates for future manufacturing. A diverse range of protein and polysaccharide biopolymers, including cellulose, corn zein, silk, and gelatin, has been utilized for this purpose. Additionally, biopolymers offer flexibility, as they can be combined with other polymers or functional agents, such as pharmaceuticals, to enhance their properties.

Among the techniques available for producing biopolymer-based nanofibers, air jet spinning has been shown to be especially effective. Despite its relatively recent development, air jet spinning enables faster and more cost-efficient fiber production, achieving comparable control and material strength to other, more established methods. Importantly, this technology enhances operational safety by eliminating the high-voltage requirements common in traditional electrospinning processes. The scalability of air jet spinning also allows for applications using an airbrush device, facilitating greater mobility and enabling direct coating of target surfaces with molded fibers. Despite higher energy consumption, air jet spinning (AJS) offers lower operational costs than electrospinning, with advancements in nozzle designs and automation improving fiber consistency, scalability, and applicability in fields like tissue engineering and drug delivery [119,120,121].

The biomedical potential of air jet-spun biopolymer nanofibers is considerable. The ability to precisely control fiber porosity, chemical composition, and structural stability renders this technique particularly suitable for medical applications, with tissue engineering being one of the most extensively studied areas. Protein and polysaccharide nanofibers serve as excellent scaffolds for cell growth and tissue repair, combining biocompatibility and tensile strength with a three-dimensional matrix structure that mimics the extracellular matrix. For drug delivery, air jet-spun fibers enable controlled release due to their stability and customizable fiber diameters. Beyond medical applications, these fibers show promise in fields such as food packaging, where they can provide antibacterial properties, air filtration through adjustable fiber porosity, and biosensor technologies. As the demand for renewable resources and sustainable materials continues to rise, biopolymer nanofibers are likely to be increasingly explored for their environmental benefits and cost-efficiency. The high level of control over fiber structure and properties also facilitates the replication of in vivo conditions, positioning air jet-spun biopolymer-based nanofibers as a promising foundation for advancing next-generation medical technologies.

## Figures and Tables

**Table 2:** Overview of materials utilized for nanofiber production via air jet spinning, detailing pressure conditions, pretreatment methods, post-treatment methods, and biomedical applications.

Materials	Pressure Used	Pretreatment Methods	Post-Treatment Methods	Biomedical Applications
Fish sarcoplasmic protein	60 psi [61]	Polymer blending with biopolymers [147]	Cross-linking with dialdehyde starch [148]	Tissue Engineering [61], Air Filtration [61]
Gelatin	60 psi [149], 0.1 MPa [150], 345 kPa [151]	Multi fluid mixing with other biopolymers like pullulan [152]	Sequential solution blow spinning-improved mechanical properties and hydrophobicity [113]	Tissue Engineering [149,150], Food Packaging [153]
Collagen	0.1 MPa [150], 0.2 bars [43]	Bilayer collagen fibers through solution blow spinning to make Nerve guidance conduits [154], PCL/collagen blends for skin regeneration [43]	Cross-linking with 1-ethyl-3-(3-dimethyl aminopropyl) carbodiimide (EDC), N-hydroxy succinimide (NHS) and Glutaraldehyde [155]	Tissue Engineering [43,150]
Silk	40 psi (0.28 MPa) [31], 80 psi [156]	Blended with soy for enhancing wound healing properties and tissue regeneration [77]	Ultrasound-assisted air-jet spinning for different structure and properties of fibers [77]	Tissue Engineering [31], Drug Delivery [156]
Corn zein protein	0.1 MPa [49], 80 psi [111], 100 psi [157]	Blending with gelatin and anthocyanin extract for intelligent food packaging [158], blending with sodium citrate for topical drug delivery [157]	N/A	Food Packaging [153], Drug Delivery [111,157]
Chitosan	10–14 psi [159], 103.42 kPa [160]	Blending with other biopolymers [161]	Air heated solution blow spinning [161]	Tissue Engineering [159,160]
Cellulose	2–5.5 bar [162], 0.5 bar [97]	Dissolving in green solvents like ionic liquids to improve dissolution of cellulose [163]	N/A	Air Filtration [162], Biosensors [97]
PLA	420 kPa [164], 2.4 kPa [33]	Blended with chitosan [161], blended with hydroxyapatite for bone tissue engineering [164]	Surface modification of nanofibrous mats to improve cell proliferation [150]	Tissue Engineering [164], Drug Delivery [33]
